# Reducing Sepsis Deaths in Newborns Through Home Visitation and Active Case Detection: Is it Realistic?

**DOI:** 10.9745/GHSP-D-17-00201

**Published:** 2017-06-27

**Authors:** Stephen Hodgins, Robert McPherson

**Affiliations:** a Deputy Editor-in-Chief, Global Health: Science and Practice Journal, Baltimore, MD, USA.; b Save the Children, Washington, DC, USA.

## Abstract

Severe bacterial infection remains one of the major causes of newborn deaths in low-income countries. A key challenge for reducing this burden is making definitive treatment more easily available. Active case detection through early postnatal home visits can work under trial conditions but is difficult to implement at scale under routine conditions. In many settings, making treatment available at peripheral-level primary health care facilities may be more feasible.

See related article by Hailegebriel.

## THE ISSUE OF SERIOUS NEWBORN INFECTION

Serious bacterial infection remains 1 of the 3 leading causes of newborn deaths globally^1^ and in some high-burden settings accounts for more than a third of such deaths. Reducing this burden requires strategies that result in more timely case identification and initiation of suitable antibiotic treatment. In many low-income, high-burden settings, achieving such improvements requires services to be pushed out more peripherally to make them more easily accessible. This is particularly challenging in places where much of the population does not currently have easy access to hospital-based care.

## EARLIER LANDMARK STUDIES

Bang et al. (1999)^2^—working in a poorly served, comparatively remote area of India—piloted an approach to reduce newborn mortality that relied on community health workers (CHWs) to provide postnatal home visits, with an intensive, closely monitored, 7-visit schedule over the first month of life. These CHWs were to identify and treat cases of possible sepsis, using oral cotrimoxazole and intramuscular gentamicin. The package also included having the CHWs assist traditional birth attendants at childbirth, resuscitating any newborns not spontaneously initiating breathing at birth. This quasi-experimental study achieved greater than 60% reduction in newborn deaths. These findings challenged a fatalistic attitude then widespread in the global health community, which assumed that important progress in reducing newborn mortality would not be possible without wide access to sophisticated hospital-based services.

Almost a decade later, in 2008 Baqui and colleagues published the results of a comparably important study,^3^ testing a similar approach in rural Bangladesh, using a cluster-randomized control trial (RCT) design with a much larger sample than in the Bang study. Like the earlier study, this trial recruited and trained its own CHWs to provide this package of services, and in addition to active case detection and treatment of possible sepsis, the intervention included CHW counseling for women and household members on essential newborn care and danger signs. It also included a community mobilization component. However, the package of interventions did not include resuscitation of non-breathing newborns. The schedule of home visits was less intensive than in the Bang study (2 visits during pregnancy, 3 in the first week of life), and the trial was implemented in a less isolated setting, where treatment services were more readily available than in the Bang study setting. The Baqui trial achieved 34% lower mortality in the intervention than the comparison arm. The findings of this study drew considerable attention, including its recognition as the *Lancet* “paper of the year” in 2008.

## WHO/UNICEF RECOMMENDATION

On the strength of these 2 studies, along with several others that didn't include a sepsis treatment component, in 2009 the World Health Organization (WHO) and the United Nations Children's Fund (UNICEF) issued a joint statement recommending introduction of postnatal home visitation by health professionals or CHWs, with assessment for danger signs and counseling on essential newborn care practices.^4^ More recently published papers^5^^,^^6^ report on a similar approach entailing active detection of cases of possible sepsis, through an intensive schedule of home visits with referral to the most peripheral level of the primary health care system for outpatient antibiotic treatment for cases for which hospital referral is not feasible. These studies demonstrated equivalent outcomes for simplified antibiotic regimens, in comparison with 7 days of injections of procaine penicillin and gentamicin, given on an outpatient basis.

WHO and UNICEF issues a joint statement in 2009 recommending introduction of postnatal home visitation, with assessment for danger signs and counseling on essential newborn care practices.

## THE CURRENT STUDY

The study by Hailegebriel and colleagues, reported in this issue of GHSP,^7^ is a useful additional piece of evidence that can inform the development of more effective strategies to reduce the population burden of preventable infection deaths among newborns. In this cluster RCT, home visitation (3 visits during pregnancy and 5 postnatally) was introduced in both intervention and control arms. One of the pregnancy visits and 2 of the postnatal visits were to be done by paid, government health auxiliaries (Health Extension Workers, or HEWs); the remainder of the visits were to be done by community volunteers, 3,500 of whom were recruited and trained for the trial. Home visits were to focus on counseling on essential newborn care practices and assessment for danger signs. Any identified cases of possible sepsis were to be referred. In the intervention arm, outpatient antibiotic treatment was made available at the health post level, provided by HEWs, if caregivers of the sick newborn were unable or unwilling to go to a higher-level facility. The intervention also included monthly review meetings with HEWs.

### Difficulty Delivering Home Visitation Even in This Trial Setting

During the initial months of the trial, although most newborns received at least some postnatal home visits, the number of cases of possible sepsis identified and treated was low. Formative research was conducted to determine barriers to care seeking, and the intervention was modified to incorporate community mobilization activities in intervention communities, following which there was a marked increase in the number of cases treated. However, even with this increase, the number of cases treated came to only about half the number expected. Furthermore, over the final 2 quarters of the intervention period, home visitation and number of cases treated tapered off. So, despite a level of support considerably exceeding what would be possible under routine conditions at scale, it was difficult to achieve and sustain adequate home visitation coverage and volume of care seeking for possible severe bacterial infection.

Despite considerable support during the trial, it was difficult to achieve and sustain adequate home visitation coverage and volume of care seeking for possible newborn infection.

A consequence of low numbers of cases identified for the trial was that it had inadequate statistical power to detect the effect size anticipated at the time the study was planned. Failing to show a statistically significant difference between intervention and control arms on the primary endpoint of the trial (day 2–27 neonatal mortality) means that the study does not provide compelling evidence for mortality-reduction effectiveness. However, neither does it provide evidence for lack of effectiveness. The measured effect size was compatible with chance (adjusted risk ratio [RR] 0.83, *P*=.33 per cluster-level analysis; RR 0.72, *P*=.09 per secondary, individual-level analysis) but also compatible with a mortality effect of the magnitude anticipated at the time of the study design, given that only about 50% of expected cases were reached. Lower than expected utilization resulted in inadequate statistical power. But this problem reflects the real-world challenges in attempting to implement such a strategy and cuts to the heart of our concern with postnatal home visitation as a strategy to reduce newborn mortality.

There is evidence (e.g., from the Bang^2^ and Baqui^3^ studies) that early postnatal home visitation can be an effective way to reach mothers and newborns with interventions that can improve outcomes, but—as results of the Hailegebriel^7^ study demonstrate—this is not easy. Key challenges with such an approach include ensuring that home visits actually happen early, at sustained, high coverage, and ensuring delivery of effective content (counseling, case detection, referral/treatment). This could be summarized as ensuring high effective coverage. Doing so requires adequately intensive inputs and program quality assurance.

In response to the 2009 WHO/UNICEF Joint Statement,^4^ a number of countries have made efforts to implement postnatal home visitation under routine public-sector program conditions. In almost all instances, countries have been unable to achieve high coverage of early postnatal home visitation.^8^ Home visitation by CHWs may seem like a simple, low-tech approach, but achieving high coverage and making sure that what happens during these contacts contributes to better outcomes takes considerable program effort. Even in the context of these trials, this was challenging. For national programs run under routine conditions, in most low- and middle-income settings this is too demanding to be feasible.

Home visitation by CHWs may seem like a simple, low-tech approach to improve maternal and newborn outcomes, but doing it successfully takes considerable program effort.

### The Self-Referral Alternative

By initial design, the primary means of identifying and ensuring early initiation of treatment for possible severe bacterial infection in the Hailegebriel^7^ trial was home visitation and active case detection. However, the study found that over time self-referral made up an increasing proportion of cases treated, and by the end of the intervention period accounted for the majority of cases. It appears that, with reliable provision of such treatment at the health post, those requiring this service were increasingly motivated to seek care, without the need for case detection during home visits. This is an encouraging sign.

The Government of Ethiopia is now moving forward to scale up provision of treatment for possible severe bacterial infection at the health post level. As such care at the health post level is being rolled out across Ethiopia, it is relying primarily on self-referral of cases rather than active case detection based on home visitation, as done under the trial. This was a sound move, given the practical difficulties with a strategy requiring active case detection.

**In every setting, health sector planners and policy makers need to make a realistic determination of the circumstances in their settings, adopting and adapting strategies most likely to be feasible and effective under real-world conditions.**^9^
